# Clinical Reports of Diseases, Injuries, Surgical Operations, Etc., Etc., at the Buffalo Hospital of the Sisters of Charity

**Published:** 1857-08

**Authors:** Benj. H. Lemon

**Affiliations:** Formerly House Surgeon


ART. V.— *Clinical Reports of Diseases, Injuries, Surgical Operations, &c., &c., in the Male and Female General Surgical and Private Wards of the Buffalo Hospital of the Sisters of Charity.* Care of Prof. HAMILTON, for the winter half year commencing Oct. 1st, 1856. By BENJ. H. LEMON, M. D., formerly House Surgeon.

(Continued from Page 102.)

CASE V.— *Uncomplicated, Idiopathic, Congenital Capsulo-Lenticular Cataract of both Eyes. Operation on one. Good result.*

Drusilla H., aged 6, a sprightly child, with fair complexion, light hair, and blue eyes. Admitted with her parents Oct. 13th, to a private ward.

The peculiarity in her eyes was first observed when she was three months old. She has never been able to go about without guidance. Has cataract of both eyes; can distinguish bright colors when not in too strong a light; the irides are normally sensitive; vision seems best in the right eye.

The solution of atropia having been dropped into the eye and applied to the brow, caused dilatation of the iris in a short time; this improved the sight. Nothing contraindicating, Dr. Hamilton determined to make the posterior operation for laceration of the capsule, and reclination of the crystalline lens. He accordingly—the iris being well dilated—introduced the needle through the sclerotic at a point two lines from the circumference of the cornea, and one below the equator, or transverse median line of the globe, carrying it through the posterior chamber until the point of the instrument came to the centre of the cataract, then lacerating and comminuting the capsule, and finishing the operation by reclining the lens within the vitreous humor. The cataract was soft, and kept the position in which it was placed. The patient was now able for the first time to see the features of her parents; she expressed no astonishment, but the medical students being present, said "she saw men."

Ordered to be kept quiet in a moderately dark room, muslin clothes saturated with cool water, to be constantly applied to the brows.

With the exception of some redness of the conjunctiva, nothing to be deprecated occurred after the operation; subsequently when sufficient time had elapsed, vision was found to be comparatively good, and available for ordinary purposes.

It was thought proper to defer the operation on the right eye that the first might recover; the shock to the system being much less, and the risk of the sympathetic influence for evil of the one organ on the other being avoided; and to operate on the worst of the two that data might be gathered which would serve as guides for the second; as in a first operation there may be discovered some constitutional peculiarity of bad tendency which may be in some measure partially or entirely avoided, or another mode of operating be thought most suitable to the peculiar circumstances of the case.

"Cataract may exist at all periods of life, no age being exempt, from the fœtus in utero to that beyond the natural limits of man's existence; it appears to belong, as an idiopathic affection, more properly to infancy, when it is mostly capsulo-lenticular; and to advanced years when it is lenticular. Of the exact nature of the change that produces the opacity, and the processes that regulate it we know little; idiopathic lenticular cataract cannot be accounted for, pathology and physiology do not afford any elucidation."—*Walton's Operative Ophthalmic Surgery.*

"For the performance of reclination the curved needle is alone required. It is indispensable that the pupil be fully dilated. An anterior operation through the cornea has been proposed and adopted. but is most inappropriate; it is only by passing the instrument through the sclerotic that sufficient command can be obtained for proper displacement.—*Ibid.*

"It is a grave objection to displacement that in performing it the interior of a healthy eye must necessarily be considerably damaged. No condition of the vitreous humor can be said to be favorable to the operation. When from morbid change it yields readily to pressure there is danger of the cataract gravitating and resting on the retina; or floating about, and producing constant annoyance by temporarily interrupting vision; besides it is then peculiarly liable to be dislocated through the pupil.—*Ibid.*

Mr. Walton's work gives no statistics of the cures effected in various kinds of cataract by the several modes of operating; but the American editor in a note says, "From a statement of the operations by sclerotic puncturation in Wills' Hospital, it appears that the cures constitute about four-fifths of the whole number of cases operated upon in that institution."

CASE VI.—*Lenticular Cataract of the Right Eye. Complicated with Amaurosis. Complete loss of vision in the left from Atresia Pupillæ. Operation, by reclination on the right eye, partially successful.*

Joseph Morgan, æt. 20, Irish; of morbid, plethoric habits. Admitted to ophthalmic ward, January 2d.

Vision of the left eye began to fail fourteen years ago. Muscæ volitantes being a prominent symptom for a long time, the sight grew worse gradually until inflammation of the whole eye and occlusion of the pupil took place as a consequence; and vision has been lost in this organ for some years. The iris being adherent to the cornea and the almost certainly existing amaurosis and cataract, forbid any surgical interference. The right eye has been available for ordinary purposes, until four months ago, from that time the sight has rapidly failed until the present, and he is now unable to walk without being guided, and can only distinguish daylight from darkness. He can see the shadow of the heavy part of the window frame on a bright day, but fails entirely to see a chaplet of bright colored flowers hanging on the wall. The symptoms being "a dirty white film, interspersed with dancing black specks, over all objects, corruscations of light, pains, &c."

There was found to be lenticular cataract, complicated with amaurosis and conjunctivitis, the eye seeming to have a tendency to inflammation. The iris is healthy and normally sensitive to light and belladonna.

In view of the existing complication it was decided not to operate, as removing the lens would only lead to very temporary benefit, if not altogether useless; but to resort to snch remedies as would improve his general health.

Feb. His condition is much improved; the inflammation has disappeared, but vision has not gained at all with his general health.

At the repeated urgent solicitations of the patient to have an effort made to restore his sight, Dr. Hamilton consented to operate.

The iris being well dilated by the application of sol. atropia, the sclerotic was punctured one and a-half lines from the edge of the cornea, in the transverse diameter of the globe.

The lower border of the upper portion of the iris was adherent to the capsule; the needle was carried through the posterior chamber to this point, and the iris detached; then lacerating the capsule the operation was terminated by reclination of the lens within the vitreous humor, the cataract maintaining the position in which it was placed.

Scarcely any change in vision took place, amaurosis being as complete as anticipated.

Directed to stay in a darkened room; cloths saturated with cool water to the forehead; feet warm.

Seven days after operation on trying his eye, thinks the sight is improved, as he can see the small bars in the window, but cannot distinguish the different colors of the flowers on the wall. The tenth day iritis supervened. The solution of atropia to keep the pupil constantly dilated; inflammation soon disappeared.

He remained in the house a long time, but neither his general health, nor his eyesight continued to improve.

On leaving the hospital the sight was in the state last mentioned, and little expectation entertained that it would ever be any better.

"In amaurosis the movements of the iris are generally impaired or lost, directly that vision is affected; and dilatation of the pupil with irregularity is common. Yet in some cases of total blindness, where the disease lies in the brain or behind the retina, the pupil may act as in health."— *Walton's Operative Ophthalmic Surgery.*

"Richter and Baron Wenzel, make mention of these peculiarities, and the latter refers the phenomenon to the iris deriving its nerves wholly from the "*lenticular or ciliary ganglion*," while the immediate organ of sight is constituted entirely by another distinct nerve.* Hence motion of the iris is not an infallible criterion as some authors have stated, that the retina is endued with sensibility."— *Samuel Cooper's Dic.*

"Although it is impossible, in particular cases, to say with certainty whether the faintness of the perception of light, be owing to the intensity of the opacity of the cataract, or to a diseased retina, still, generally speaking, the degree of vision is in proportion to the opacity of the pupil, and its amount affords a pretty sure index of the health of that tunic."— *Walton.*

"The capacity of distinguishing light from darkness, and in a shady place where the pupil is not too much contracted, of perceiving bright colors, and

* Vide Carpenter's Principles of Human Physiology, pp. 697 and 831, sections 724 and 847. For nerves that influence contraction and dilatation of the *iris*.

the shadows of objects, is as Scarpa has particularly noticed, a very important desideratum in every case selected for operation." — *Cooper.*

"A body, such as a penknife, or the finger, should be passed between the patient's eye and the light, as a test of sight; if he perceives it, the retina may be considered sound, but unhealthy in proportion as only larger bodies can be discerned.

"An operation, when it evinces feebleness, must ever be attendant with doubtful results. With total loss of power, any practical surgical proceeding is inadmissible." — *Walton.*

"The concurrent testimony of almost all writers upon the subject, tends to prove that the restoration of sight has sometimes been effected in the most hopeless cases; and I am, therefore, of opinion with Mr. Lucas, that in all doubtful cases an operation should be tried, as a remedy by no means hazardous or violent." — *Cooper.*

*Remarks.* — From the foregoing quotations it may be inferred that amaurosis and cataract, in the incipient stage, when coëxistent in the same organ, would cause a greater degree of blindness than either singly, when in an advanced stage of the disease. The former, seeking a strong light, because the unsound retina requires a greater impression than in health. The latter, preferring the shade, that the iris may be dilated, and thus light admitted at the circumference of the cataract, the opacity of the crystalline lens being the most intense at its central point, strong light would stimulate the pupil to greater contraction, thus increasing the blindness.

These diseases, therefore, when existing together in the same organ, even in a slight degree, would tend to cause a great amount of blindness; that which is beneficial to either, when present singly, becomes detrimental to both when the complication exists.

